# Multisociety endorsement of the 2024 European guideline recommendations on coronary revascularization

**DOI:** 10.21470/1678-9741-2025-0900

**Published:** 2025-01-20

**Authors:** Victor Dayan, Joseph F. Sabik III, Minoru Ono, Marc Ruel, Song Wan, Lars G. Svensson, Leonard N. Girardi, Y. Joseph Woo, Vinay Badhwar, Marc R. Moon, Wilson Y. Szeto, Vinod H. Thourani, Rui M. S. Almeida, Zhe Zheng, Walter J. Gomes, Dawn S. Hui, Rosemary F. Kelly, Miguel Sousa Uva, Joanna Chikwe, Faisal G. Bakaeen

**Affiliations:** 1 Centro Cardiovascular Universitario and Instituto Nacional de Cirugia Cardiaca, Hospital de Clinicas, Universidad de la Republica, Montevideo, Uruguay; 2 Department of Surgery, University Hospitals Cleveland Medical Center, Case Western Reserve University, Cleveland, Ohio, United States; 3 Department of Cardiac Surgery, The University of Tokyo, Tokyo, Japan; 4 Division of Cardiac Surgery, University of Ottawa, Ottawa, Ontario, Canada; 5 Institute of Cardiovascular Surgery, Sino-Swiss Heart-Lung Transplantation Institute, Tongji Hospital, Tongji Medical College, Huazhong University of Science and Technology, Wuhan, China; 6 Coronary Center, Department of Thoracic and Cardiovascular Surgery, Miller Family Heart, Vascular, & Thoracic Institute, Cleveland Clinic, Cleveland, Ohio, United States; 7 Department of Cardiothoracic Surgery, New York Presbyterian/Weill Cornell Medical Center, New York, NY, United States; 8 Department of Cardiothoracic Surgery, Stanford University School of Medicine, Stanford, Calif; 9 Department of Cardiovascular and Thoracic Surgery, WVU Heart and Vascular Institute, West Virginia University, Morgantown, WV, United States; 10 Division of Cardiothoracic Surgery, Baylor College of Medicine, Houston, Texas, United States; 11 Division of Cardiovascular Surgery, Department of Surgery, Perelman School of Medicine, University of Pennsylvania, Philadelphia, PA, United States; 12 Department of Cardiac Surgery, Piedmont Heart Institute, Atlanta, GA, United States; 13 Instituto de Circurgia Cardiovascular, Faculdade de Medicina, Centro Universitário Fundação Assis Gurgacz, Cascavel, Paraná, Brazil; 14 Department of Cardiovascular Surgery, Fuwai Hospital, National Center for Cardiovascular Diseases, Chinese Academy of Medical Sciences and Peking Union Medical College, Beijing, China; 15 Cardiovascular Surgery, Pirajussara Hospital, Hospital São Paulo, Escola Paulista de Medicina, Universidade Federal de São Paulo (UNIFESP), São Paulo, Brazil; 16 Department of Cardiothoracic Surgery, University of Texas Health Science Center, San Antonio, Texas; 17 Division of Cardiothoracic Surgery, University of Minnesota, Minneapolis, Minn, United States; 18 Department of Cardiothoracic Surgery, Hospital de Santa Cruz, Carnaxide, Portugal; 19 Department of Cardiac Surgery, Cedars-Sinai Medical Center, Smidt Heart Institute, Los Angeles, Calif, United States

**Keywords:** CABG, coronary artery bypass grafting, coronary artery disease, CAD, coronary guidelines, left ventricular ejection fraction, optimal medical therapy, OMT, percutaneous coronary intervention, PCI

The European Society of Cardiology (ESC) recently published their 2024 guidelines for the management of chronic coronary syndromes^[1]^. This was a collaborative multidisciplinary document authored by 28 experts from 13 countries in addition to the ESC Scientific Document Group. The document was reviewed by 43 experts from 22 countries. It received official endorsement by the European Association for Cardio-Thoracic Surgery.

The ESC document is based on the best-available evidence and provides an important and timely data-driven correction to the recent course of events in the coronary guideline arena.

***The surgical societies represented in this statement endorse the recommendations of the 2024 ESC guidelines for the management of chronic coronary syndromes.***

Historically, the American Association for Thoracic Surgery (AATS) and the Society of Thoracic Surgeons (STS) have worked closely with the American College of Cardiology (ACC) and the American Heart Association (AHA) on coronary and other cardiovascular guideline documents. This collaboration was created on the basis of mutual respect and a joint, rigorous scientific commitment with a common overarching goal of developing robust and high-quality guidelines that translate to improved patient care. However, in 2021, a disruption of this longstanding collaboration (hoped to be a temporary aberration) took place, with the ACC, AHA, and the Society of Cardiovascular Angiography & Interventions publishing their guideline for coronary revascularization without the endorsement of the AATS and STS^[2]^. Notably, the AATS and STS had identified significant issues relating to the scientific accuracy of some of the recommendations pertaining to coronary artery bypass grafting (CABG) and raised those during the development and review phases of the document, but unfortunately they were not addressed^[3]^. Professional cardiovascular societies from across the globe issued individual statements that echoed the concerns of AATS and STS^[4^,^5^,^6]^.

**Central Message:**
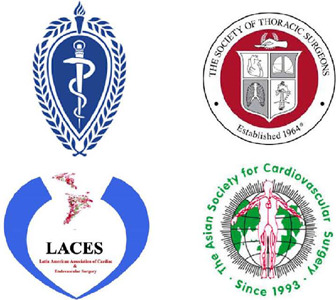
A critical review of the best-available evidence produces trustable, internationally endorsed coronary guidelines. Multidisciplinary collaboration is instrumental in guiding patient care.

Central to these concerns was a downgrade in the class of recommendation, from I to IIb, for CABG as a treatment to improve survival in patients with stable 3-vessel coronary artery disease (CAD), preserved left ventricular function, and no left main coronary artery stenosis. This downgrade was not supported by meaningful or relevant data and discounted previous well-established longitudinal evidence^[7]^. Subsequently, the 2023 AHA/ACC Guideline for the Management of Patients with Chronic Coronary Disease turned into a missed opportunity to move beyond the shortfalls of the 2021 guidelines^[8^,^9]^. The arguments of the worldwide critique of the ACC/AHA guidelines related to the indication for and the mode of revascularization for chronic CAD. The key aspects were these as follows:

- Diminishing the significance of the evidence supporting the survival benefit of CABG *versus* optimal medical therapy (OMT) alone.
- Disregarding the evidence for improved survival after CABG *versus* percutaneous coronary intervention (PCI) in patients with complex 3-vessel disease.
- Using the International Study of Comparative Health Effectiveness with Medical and Invasive Approaches (ISCHEMIA) study findings on a strategy of initial invasive *versus* conservative management of chronic coronary disease and inappropriately extrapolating them to compare CABG *versus* OMT. As is well known, the latter was not a randomized comparison in the ISCHEMIA trial.
- Applying results from revascularization meta-analyses that focused primarily on PCI *versus* OMT in lower risk patients in order to compare CABG *versus* OMT.
- Departing from a Heart Team approach in writing guidelines.

The 2024 ESC guidelines for managing chronic coronary syndromes provide a thoughtful perspective that aligns with the scientific arguments and considerations raised by multiple global professional societies.

Regarding the indication for revascularization in patients with 3-vessel disease, the 2024 ESC guidelines state: “In chronic coronary syndrome (CCS) patients with left ventricular ejection fraction >35%, myocardial revascularization is recommended, in addition to guideline-directed medical therapy, for patients with functionally significant 3-vessel disease to improve long-term survival and to reduce long-term cardiovascular mortality and the risk of spontaneous myocardial infarction” (class I, level of evidence A). Notably, the document denotes the consistent reporting of higher repeat revascularization rates with PCI independent of multivessel CAD anatomic severity.

Regarding the mode of revascularization, CABG and OMT is recommended over both PCI and OMT alone for patients with diabetes (class I, level of evidence A). In patients without diabetes, CABG is recommended over OMT alone to improve survival, symptoms, and major cardiovascular events (class I, level of evidence A). PCI is recommended along with CABG in patients with intermediate or low coronary complexity only if similar completeness in revascularization (compared with CABG) can be achieved (class I, level of evidence A). The justification in this scenario is that PCI is a less-invasive option that is noninferior in overall survival. CABG, however, is superior to PCI in reducing spontaneous myocardial infarction and cardiovascular death in the latter cohort. A major new recommendation is that when PCI and CABG have equal recommendations, a Heart Team discussion is needed and ad hoc PCI should not be performed (class I, level of evidence C).

We acknowledge the paucity of modern-day evidence on comparative effectiveness of CABG *versus* OMT, especially in patients with preserved left ventricular ejection fraction, while recognizing that the lack of equipoise in patients with severe multivessel CAD will make a randomized trial difficult if not impossible to do. However, contemporary evidence does provide reassuring data for the safety of initial medical management in patients with low atherosclerotic burden with close follow-up and future revascularization as clinically indicated^[7]^.

In summary, until new evidence changes our current assessment, the surgical societies represented in this statement support the recommendations of the 2024 ESC guidelines for the management of chronic coronary syndromes. The consensus is that in patients with complex 3-vessel CAD on OMT, CABG is recommended to improve survival and decrease major adverse cardiovascular events and symptoms (compared with OMT alone or PCI), irrespective of left ventricular ejection fraction. The patient risk profile, Heart Team discussions, and informed patient preferences are all important qualifiers in the decision-making process.

Finally, the cardiothoracic surgical societies remain committed to future collaboration with our colleagues from various disciplines for the benefit of our patients and the betterment of our field. The importance of this collaboration was emphasized in a joint society Guideline Methodology Manual^[10]^ based on the Institute of Medicine principles of trustworthy guidelines^[11]^. The Manual underscores the importance of fair representation on guideline writing and review committees and the use of a validated consensus-building process. It provides a framework that prioritizes transparency and safeguards against bias. Through adherence to these fundamental principles and a return to a Heart Team approach, it is sincerely hoped that all multidisciplinary specialty cardiovascular societies can once again align on future guideline documents for the common purpose of bettering the care of our patients.
